# In Silico and In Vitro Analyses Reveal Promising Antimicrobial Peptides from Myxobacteria

**DOI:** 10.1007/s12602-022-10036-4

**Published:** 2022-12-31

**Authors:** Benita S. Arakal, David E. Whitworth, Philip E. James, Richard Rowlands, Neethu P. T. Madhusoodanan, Malvika R. Baijoo, Paul G. Livingstone

**Affiliations:** 1 School of Health Sciences, Cardiff Metropolitan University, Cardiff, UK; 2 Department of Life Sciences, Aberystwyth University, Wales, UK

**Keywords:** Antimicrobial peptides, Myxobacteria, Biosynthetic gene clusters, In silico analysis

## Abstract

Antimicrobial resistance (AMR) is a global concern, and as soon as new antibiotics are introduced, resistance to those agents emerges. Therefore, there is an increased appetite for alternative antimicrobial agents to traditional antibiotics. Here, we used in silico methods to investigate potential antimicrobial peptides (AMPs) from predatory myxobacteria. Six hundred seventy-two potential AMP sequences were extracted from eight complete myxobacterial genomes. Most putative AMPs were predicted to be active against *Klebsiella pneumoniae* with least activity being predicted against *Staphylococcus aureus*. One hundred seventeen AMPs (defined here as ‘potent putative AMPs’) were predicted to have very good activity against more than two bacterial pathogens, and these were characterized further in silico. All potent putative AMPs were predicted to have anti-inflammatory and antifungal properties, but none was predicted to be active against viruses. Twenty six (22%) of them were predicted to be hemolytic to human erythrocytes, five were predicted to have anticancer properties, and 56 (47%) were predicted to be biofilm active. In vitro assays using four synthesized AMPs showed high MIC values (e.g. So_ce_56_913 250 µg/ml and Coral_AMP411 125 µg/ml against *E. coli*). However, antibiofilm assays showed a substantial reduction in numbers (e.g. Coral_AMP411 and Myxo_mac104 showed a 69% and 73% reduction, respectively, at the lowest concentration against *E. coli*) compared to traditional antibiotics. Fourteen putative AMPs had high sequence similarity to proteins which were functionally associated with proteins of known function. The myxobacterial genomes also possessed a variety of biosynthetic gene clusters (BGCs) that can encode antimicrobial secondary metabolites, but their numbers did not correlate with those of the AMPs. We suggest that AMPs from myxobacteria are a promising source of novel antimicrobial agents with a plethora of biological properties.

**Supplementary Information:**

The online version contains supplementary material available at 10.1007/s12602-022-10036-4.

## Introduction

Antimicrobial resistance (AMR) is a hidden pandemic with exponential increases in infections claiming lives, hospitalizations, and financial burdens. Recent reports suggest that by 2050 ten million people will die annually due to AMR, which is a huge public health concern [1]. When antibiotics were introduced, reports of resistance followed immediately. In recent times, diminishing natural product discoveries and inefficacious chemical modification of existing antibiotics to create next-generation derivatives have dissuaded pharmaceutical companies from such high-risk investments and have resulted in a global crisis [2]. This has led to interest in alternatives to traditional antibiotics, such as immunotherapeutics, bacteriophages, probiotics, and antimicrobial peptides (AMPs) [3]. AMPs are short protein sequences that are ubiquitously produced on ribosomes by all kingdoms of life [4]. Also referred to as host defense peptides in eukaryotes, AMPs constitute an integral part of the immune system as they exhibit immunomodulatory functions which stimulate chemokine production and signalling cascades of pro-inflammatory and anti-inflammatory responses [5]. AMPs are also capable of enhancing phagocytosis, possess anticancer properties, increase angiogenesis by recruiting immune cells at inflammation sites, and can promote wound healing [6, 7]. However, what makes these biomolecules noteworthy in the context of AMR are their antibacterial, antifungal, antiviral and anticancer properties [6].

Similarly to eukaryotes, bacteria also produce ribosomally synthesized AMPs which have microbicidal effects, by either damaging bacterial membranes followed by release of cellular contents or by formation of transient pores in bacterial membranes, therefore resulting in cell lysis [8]. A classic example of bacterial AMPs which exhibit such mechanisms of action are ribosomally synthesized bacteriocins. Bacteriocins are heterogenous peptides whose physical characteristics and killing strategies are varied between Gram-positive and Gram-negative bacterial species [9]. Microcin J25, a Gram-negative bacteriocin derived from *Escherichia coli*, is a highly stable post-translationally modified peptide and has been shown to possess antibacterial activities against *Shigella, E. coli*, and more recently, drug-resistant *Salmonella* [10]. Gram-positive bacteriocins such as lysostaphins have potent antistaphylococcal effects and due their enhanced antibiofilm activity, and hydrogels formulated of this lysostaphin have eliminated methicillin-resistant *Staphylococcus aureus* when tested on infected bone fractures [11].

Myxobacteria are a phylum of soil predators which exhibit a unique multicellular lifestyle facilitated by a high degree of co-operation, predominantly characterized by fruiting-body formation, and the ability to prey broadly on a wide range of bacteria and fungi [12–14]. Understanding how myxobacteria kill their prey could bring to light novel antimicrobial molecules, including AMPs. Mining myxobacterial genomes for their biosynthetic gene clusters (BGCs) and the metabolites they produce (including non-ribosomal peptides) has provided valuable insights for natural product discovery [15, 16]. However, this approach has not yet brought any antibiotic to the market, although several are in preclinical or clinical trials. Investigations into the ribosomally synthesized AMPs of myxobacteria are still at an early stage. In the current genomics era, bioinformatics-led approaches to shortlist potential AMPs are commonly adopted, rather than laborious ‘grind and find’ experimental approaches for high-throughput screening of potential AMPs [17–19]. Moreover, in silico approaches provide the opportunity to characterize potential alterations/mutations within AMP sequences in a high-throughput fashion, developing as well as identifying putative AMPs, which can then be validated by experimental methods. This study used a suite of bioinformatics tools to identify and characterize the predicted physiochemical, antimicrobial, and functional properties of putative AMPs from eight complete genomes of myxobacteria and test four synthesized AMPs for their antimicrobial and antibiofilm properties by in vitro assays.

## Materials and Methods

### Bacterial Genomes and Antimicrobial Peptide Searches

Eight complete genomes (Table 1) from six genera of myxobacteria were included in the study. Peptides were identified using the BactPepDB database [20] screening for peptides of more than 20 amino acids located in both coding and intergenic regions. Antimicrobial activity of the peptides was predicted using the Database of Antimicrobial Activity and Structure of Peptides DBAASP.v3 [21]. The peptides were initially screened for potential AMPs using the general prediction tool (BactPepDB), which can predict linear peptides that have antibacterial activity based on their physicochemical features. Those peptides that had probable antibacterial activity were further analysed using DBAASP which predicted the activity against five bacterial species, *Escherichia coli* ATCC 25,922, *Pseudomonas aeruginosa* ATCC 27,853, *Klebsiella pneumoniae*, *Staphylococcus aureus* ATCC 25,923, and *Bacillus subtilis*. An active peptide against a particular bacterium was defined as one with a predicted minimum inhibitory concentration of < 25 ug/ml. Selected AMPs were also queried through CAMPR3 [22], a web tool that runs multiple AMP prediction models (support vector machines (SVMs), random forests (RF), and discriminant analysis (DA)).

**Table 1:** Putative AMP and BGC counts of the eight genomes analysed

**Organism**	**Genome size**	**Predicted AMPs**	**‘Potent’ predicted AMPs**	**BGCs**
*Anaeromyxobacter dehalogenans* 2CP-1	5.03 Mb	40	10	7
*Corallococcus coralloides* DSM 2259	10.08 Mb	49	5	34
*Haliangium ochraceum* DSM 14,365	9.4 Mb	144	27	24
*Myxococcus fulvus* HW-1	9 Mb	72	14	25
*Myxococcus stipitatus* DSM 14,675	10.4 Mb	64	11	27
*Myxococcus xanthus* DK 1622	9.1 Mb	82	15	23
*Sorangium cellulosum* SoCe56	13.03 Mb	114	21	36
*Stigmatella aurantiaca* DW4/3–1	10.3 Mb	107	14	39

### Physiochemical Properties and Toxicity of Active Peptides

Peptides predicted to be active by DBAASP were further analysed for their molecular weight, hydrophobicity, and total net charge using the APD3 tool [23]. The APD3 database uses 2169 antimicrobial peptides to predict various characteristic features of a query peptide. APD3 was also used to screen antiviral and antifungal properties. ToxinPred [24] was used to predict the toxicity of the peptides based on the presence of certain amino acid residues such as Cys, His, Asn, and Pro in specific positions. Haemolytic properties of the peptides were predicted using HemoPred [25], a tool that used a random forest classifier taking into account amino acid composition, dipeptide com-position, and physicochemical features. AIPpred [26] was used to predict anti-inflammatory properties using various features such as dipeptide composition, amino acid index, chain-transition-distribution, and physicochemical properties. Anticancer properties were predicted using ACPred [27], which utilizes SVM and RF machine learning models. Biofilm activity was predicted using dPABBs [28] which is based on whole amino acid composition, selected residue features, and positional preferences of residues.

### Functional Associations of Potent Antimicrobial Peptides

Peptides predicted to have activity against at least three bacteria were subjected to BLAST-P searches against the NCBI database to identify the closest sequences. Those annotated proteins from the NCBI database that had 100% query coverage and > 85% similarity to predicted AMPs were further subjected to functional analysis using the STRING database v11 [29] to identify functional association networks.

### Antimicrobial Peptides in the Myxobacterial Pangenome

Publicly available genomes (Table 1) of eight myxobacterial species belonging to six genera were downloaded from the NCBI database, and all pairwise average nucleotide identities were estimated (http://enve-omics.ce.gatech.edu/ani/) [30]. Of the eight species, only *Myxococcus xanthus* and *Corallococcus coralloides* had more than two genomes published in the NCBI database at the time of this study. For those two species, the web tool SPINE/AGEnt/ClustAGE [31] was used to carry out pangenome analysis. The NUCmer function of the integrated MUMmer software package within SPINE was used to align genomes (*Myxococcus xanthus* CA005, *Myxococcus xanthus* DK 1622, *Myxococcus xanthus* DSM 16,526, *Myxococcus xanthus* DZ2, *Myxococcus xanthus* DZF1, *Myxococcus xanthus* AB036A, *Myxococcus xanthus* AB056, *Myxococcus xanthus* KF3.2.8c11, *Myxococcus virescens* NBRC 100,334, *Myxococcus virescens* DSM 2260, *Corallococcus coralloides* DSM 2259, *Corallococcus coralloides* B035, and *Corallococcus coralloides* CA044C) and identify core genes. The SPINE-derived core genome was subtracted from individual genomes using the AGEnt tool to obtain accessory genome elements, which were clustered to acquire the minimum set of accessory genes distributed between individual genomes.

### Genome Mining for Secondary Metabolites

The eight genomes were submitted to antiSMASH 6.0 [32], a web server and standalone tool that analyses genomes for BGCs, predicting metabolites produced by unknown BGCs by comparing with those producing previously characterized secondary metabolites. Ribosomally synthesized and post-translationally modified peptides (RiPPs) and bacteriocins were analysed using the BAGEL-4 [33] web server.

### Peptide Synthesis, MIC, and Antibiofilm Properties

Four AMPs (Stig_213, Coral_AMP411, Myxo_mac104, and So_ce_56_913) were chosen to be synthesized based on their in silico physiochemical, antimicrobial, and antibiofilm properties (Tables S1 and S2). The peptides were synthesized at Peptide Protein Research Ltd., Fareham, UK, and crude desalted lyophilized peptides were diluted in sterile distilled water in 1% DMSO to get a stock solution of 1000μg/ml of each peptide. *Escherichia coli* ATCC 25,922, *Pseudomonas aeruginosa* ATCC 27,853, *Klebsiella pneumoniae* ATCC 13,883, *Staphylococcus aureus* ATCC 25,923, and *Bacillus subtilis* (wild-type laboratory strain) were used to test their MICs and antibiofilm properties. Bacterial inoculum were optimized by determining their colony forming counts (CFUs) by viable count enumeration and adjusting to 5 × 10^5^ CFU/ml. Appropriate antibiotics (Table 3) were used as positive controls in each assay. MICs were done by micro broth dilution method in 96-well polypropylene plates (Thermo Fisher Scientific, UK) as recommended by Clinical and Laboratory Standards Institute (CLSI) methods [48]. The AMPs were diluted twofold in Mueller Hinton broth (MHB) to get final concentrations ranging from 500μg to 7.8μg/ml. To the diluted AMPs, bacterial cells (8 × 10^5^) were added and incubated overnight. Negative controls of MHB without AMPs were used, and the assays were done in triplicates. After overnight incubation, MICs were deduced by the lowest concentration that showed no visible growth. Antibiofilm properties were screened with assays as described for the MICs. After incubation, unattached bacteria were removed by washing with phosphate-buffered saline (PBS—pH 7.4) (Merck Life Science UK Limited, Dorset, UK) and stained with Cell titer blue (Promega, Southampton, UK) to enumerate viable cells by spectrophotometry. To look at biofilm degradation, bacteria were grown in 96-well plates without AMPs, and after overnight incubation, unattached bacteria were removed by washing with PBS. Biofilms were then treated with AMPs, and live bacterial cells were enumerated as described above.

## Results

**Fig. 1:**
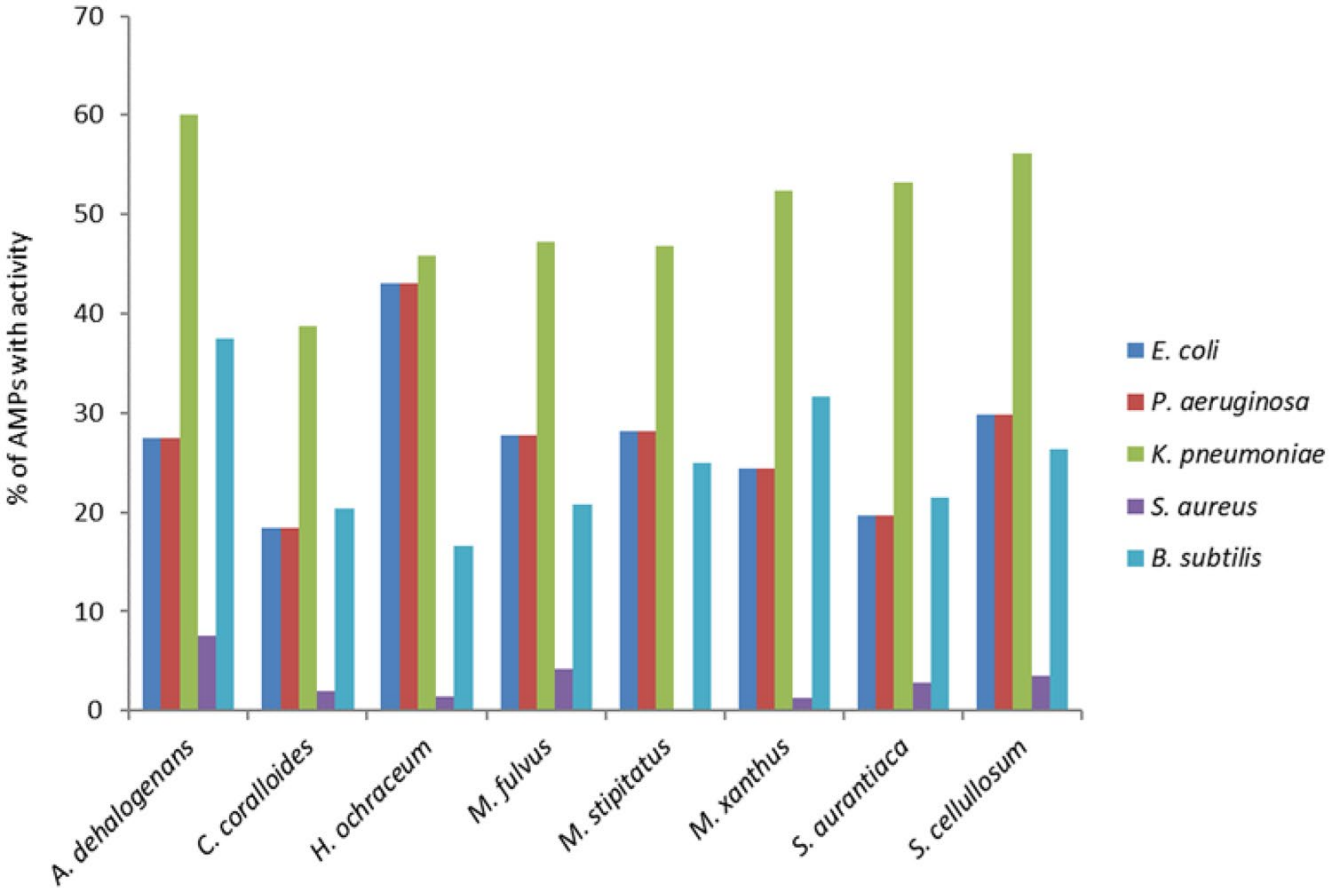
Predicted activity profile of putative AMPs from eight myxobacterial genomes, against five species of pathogens. Activity shown as the percentage of AMPs from myxobacteria with predicted activity against each pathogen

**Fig. 2:**
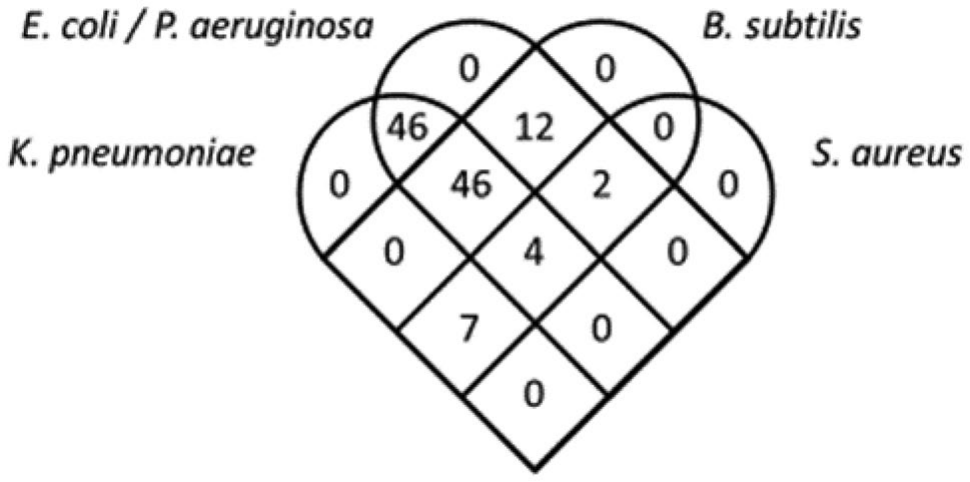
Predicted activity of the 117 potent myxobacterial AMPs against five pathogenic bacteria

A total of 672 putative AMP sequences were extracted from the eight complete genomes (Table 1) using the Bacteria Peptide Database (bactpepdb.rpbs.univ-paris-diderot.fr) (Tables S1 and S2) The peptides ranged between 20 and 80 amino acid residues in length. *Haliangium ochraceum* had the highest number of putative AMP sequences (144), while *Anaeromyxobacter dehalogenans* had the lowest with 40 predicted (Table 1). *K. pneumoniae* was predicted to be susceptible to the largest number of myxobacterial predicted AMPs, followed equally by *E. coli* and *P. aeruginosa*, and with *S. aureus* predicted to be susceptible to the lowest number of predicted AMPs (Fig. 1). One hundred seventeen putative AMPs, ranging from 20 to 74 amino acid residues in length, were predicted to be active against three or more of the five pathogens (*E. coli*, *P. aeruginosa*, *K. pneumoniae*, *S. aureus*, *and B. subtilis*) tested (defined hereafter as ‘potent’ putative AMPs) (Fig. 2). All 117 sequences were predicted by the AIPPred tool to have anti-inflammatory properties. Further filtering of the 117 ‘potent’ putative AMPs, by predicted toxicity (haemolytic and cytotoxic), net charge (> 2), hydrophobicity (> 20%), amino acid length (20–50 residues), and whether also predicted to be AMPs by CAMPR3, resulted in a shortlist of 37 highest confidence candidate AMPs (Table 2).

**Table 2:** Highest confidence putative AMPs

	**AMPs**	**Length (aa)**	**Total hydrophobic ratio**	**Total net charge**
1	Anaero2CP1_10 MHSQRATGRLLRQARELARLD	21	38%	3.25
2	Anaero2CP1_34 MRIGLARAASVVVPRHARLASFVTQ	25	56%	4.25
3	Coral_AMP336 MAREPQARFAGSGGPSIPIAARIRETPAA	29	41%	2
4	Coral_AMP411 MMGAPTRRFKHHAWHETTVARRATARYVGGLSSRFVTR	38	37%	7.75
5	Haliangium_och123 VRGERVKFPRPNSRGLIEADRLF	23	35%	3
6	Haliangium_och145 VLRTLRPILVCRFREFRTPKWTE	23	43%	4
7	Haliangium_och379 VASLKRADGGRVSWQTRLVSTTQQSWPH	28	32%	3.25
8	Haliangium_och394 MPYPKFARRIRIRSFSFRFSLCGSLDSLT	29	41%	5
9	Haliangium_och413 MTQLPRRALPPPRLVGLGALRAALLLGRWG	30	50%	5
10	Haliangium_och414 MDRVLAERQGRKKTNMQPSHLITNQPGRLL	30	30%	4.25
11	Haliangium_och424 MLACTSRETRHLPTGYHQSRSGRFGFGSTR	30	23%	4.5
12	Haliangium_och426 MPLPQRRERRGFSKPFAPEPLPALSRFDVM	30	37%	3
13	Haliangium_och530 VASLKPVVDRRIRQRLDGFPRPNSRGLIEAPRPWW	35	37%	5
14	Haliangium_och588 VFSLTSIPGALRRRLIGRSARALGALGLPSDSHWKSDS	38	39%	4.25
15	Haliangium_och694 VRQPRNPRRDRGLRPKKCRASPTGVSAAATSKAPHKLPRRRPGA	44	25%	13.25
16	Haliangium_och750 VRLAIKRVNRARRGGARTRRHAFESFAYDARGRGQLVHSVEKMLEQSF	48	38%	8.5
17	Myxo_mac104 VNRVTRVIATRRNEAERIGVPLYF	24	42%	3
18	Myxo_mac128 LEGRGPPRRRRALEVGLAKLGCYAVR	26	38%	5
19	Myxo_mac77 MARPPHLKLANTSKSNVLLSNSA	23	39%	3.25
20	Myxo_stip126 MRSVSATLCLYSNPVASRNRRLSTTMG	27	37%	4
21	Myxo_stip147 MPEGVEHFFPAFTHPPIWSTAARRGAGRR	29	38%	2.5
22	Myxo_stip166 MSRRLLVSSRRSWPRARAGVVPRFFRVVMP	30	47%	8
23	Myxo_stip248 MRCVPKQERRNQAWRLKRFTKDSTMPLLSESRLAWFG	37	38%	6
24	Myxo_stip360 MRTDARSPRRRQLRWGDVHHRPQHGLRGPQSSILRVHAPRFFQSLQRTR	49	27%	11
25	Myxo_stip99 VVTLEGKVGRAGGRAESRAVWRVSSG	26	38%	3
26	Myxo_xan108 MAPPRAVRPARDVAGGAGGGQWPWP	25	40%	2
27	Myxo_xan118 MLTLNLAQRILCRPVRIFKQVRLTQ	25	52%	5
28	Myxo_xan210 VGTAGRVTLHGVAAPLSHLATRSRLKALPVAR	32	47%	5.5
29	So_ce_56_119 MKDGAGRSVLRLMRPAAMRAHL	22	50%	4.25
30	So_ce_56_843 VIPAAPSASEPRGPLRGGAARARRGPARAARGAALPGANGRAASVRA	47	43%	8
31	So_ce_56_913 VEKSEKAISGARRGSPIVNRHVVHLEHVRLKGPYRLSDRLSSAPRTSTRV	50	30%	7.75
32	Stig_102 MSMSRGTKPSSREASARLFKRGAA	24	33%	5
33	Stig_152 VRARAREPIRRVGLGDTPTIDPVVWR	26	38%	3
34	Stig_213 VVKTVVSRAYTRAGLAQRLGWHDLRHSTRT	30	37%	5.5
35	Stig_261 MRLGGANGEGLGGPGRRLRARVREWLARIPESMT	34	35%	4
36	Stig_370 VKRFATPVHFLRKPFRKGRARLRKAIAAQIGATLSRLFPSR	41	44%	12.25
37	Stig_93 MTGLRILARNGLSLRALRQHRRS	23	39%	6.25

‘Potent’ putative AMP sequences were also BLAST-P searched (100% query cover), and top-scoring hits used to find functional associations with other proteins using the STRING database (Table S3). Thirteen putative AMPs with > 85% sequence similarity showed significant associations with a variety of other proteins. Pangenome analysis of *Myxococcus xanthus* and *Corallococcus coralloides* (for which more than two complete genomes each were available) showed > 75% AMPs were part of the accessory genome, suggesting the possibility they have been acquired by horizontal gene transfer (HGT). Genome mining for BGCs using antiSMASH predicted the production of a variety of secondary metabolites, which together with the number of AMPs predicted, confirms that myxobacteria are a potentially rich source of antimicrobial compounds (Table S4). Although there was a strong correlation between the size of the genomes and the number of BGCs (*r*-squared = 0.769), there were no correlations between the number of AMPs and either genome size or the number of BGCs (*r*-squared values < 0.3) (Table 1).

## *Anaeromyxobacter dehalogenans* 2CP-1

Compared to the other myxobacteria, *A. dehalogenans* had the lowest number of putative AMPs (40), but ten of them were predicted to have very good antibacterial activity. Anaero2CP1_175 was predicted to have antibacterial activity against all five bacteria tested, and seven peptides were predicted to be active against four pathogenic bacteria, while two of them were predicted to be active against three pathogenic bacteria. In common with other myxobacteria, the largest number of putative AMPs from *A. dehalogenans* were predicted to have activity against *K. pneumoniae*, while 5% of the AMPs were predicted to be active against *S. aureus*. Activity predictions suggested two of the ten AMPs were likely to be haemolytic to human RBCs, with three (Anaero2CP1-256, Anaero2CP1_262, and Anaero2CP1_264) having predicted anticancer properties. Six AMPs were expected to have antibiofilm properties in their native forms and one (Anaero2CP1-10) in a mutated form, with a change in the first amino acid position (Table S2).

BLAST-P analysis of the ‘potent’ *A. dehalogenans* AMP sequences found that Anaero2CP1_10 had 100% sequence similarity to a region of a tetratricopeptide repeat protein from *Enhygromyxa salina*. This protein has functional associations with several other hypothetical proteins (Table S3). Anaero2CP1_341, a 74 amino acid residue peptide, had a 100% similarity to a 74-residue hypothetical protein from *A. dehalogenans* which was functionally linked to other hypothetical proteins and transcriptional regulator proteins (Table S3). Only seven BGCs were identified in the *A. dehalogenans* genome.

## *Corallococcus coralloides* DSM 2259

The *C. coralloides* DSM 2259 genome had 49 putative AMP sequences with five of those predicted to have activity against three or more of the pathogenic bacteria tested; however, two of the five were predicted to be haemolytic to human RBCs. Three of the ‘potent’ putative AMPs were predicted to have antibiofilm properties (Table S2). Like the AMPs of other myxobacteria, those from *C. coralloides* were most frequently predicted to have activity against *K. pneumoniae*. None of the AMPs had a sequence similarity of > 85% with any annotated proteins on BLAST-P search. Thirty-four BGCs were seen in the genome using antiSMASH searches. Pangenome analysis using the three *C. coralloides* genomes available on the NCBI database showed that 12 putative AMPs were part of the core genome, with the rest in the accessory genome, presumably having been acquired through HGT.

## *Haliangium ochraceum* DSM 14,365

*H. ochraceum* had the highest number of putative AMP sequences (144) compared to other myxobacterial genomes, including 27 predicted ‘potent’ AMPs with antibacterial activity against three or more of the pathogenic bacteria tested. Haliangium_och750 was predicted to be active against five of the pathogenic bacteria tested, with another nine predicted to be active against four. Six of the ‘potent’ putative AMPs were predicted to be haemolytic, and one (Haliangium_och1055) was predicted to have anticancer properties. dPABBs software predicted 14 AMPs have antibiofilm properties in their native forms and two in mutated forms (Table S2). The set of AMPs were predicted to be almost equally active against *E. coli*, *P. aeruginosa*, and *K. pneumoniae*, but did not do well against *S. aureus*, with just Haliangium_och750 predicted to be active against it. Hali-angium_och974 and Haliangium_och996 had 100% similarity with hypothetical proteins of H. *ochraceum*. Those hypothetical proteins were functionally associated with several other hypothetical proteins (Table S3). AntiSMASH analysis of the genome showed the presence of 24 BGCs producing possibly antibacterial, antioxidant, and anticancer metabolites.

## *Myxococcus macrosporus* HW-1

*M. macrosporus* HW-1, previously known as *Myxococcus fulvus* HW-1, was recently classified based on its genomic features [34]. The genome encoded 72 putative AMP sequences with *K. pneumoniae* (37%) being the pathogen most AMPs were predicted to be active against and *S. aureus* (3%) with least activity. Fourteen putative AMPs were predicted to possess very good antibacterial activity, with three of the AMPs being active against four of the five bacteria analysed. HemoPred software predicted four putative AMPs to be haemolytic to human RBCs, and dPABBs predicted seven to be biofilm active in their native form and three in their mutated forms (Table S2). Four-predicted AMP sequences had high sequence similarity with annotated proteins on the NCBI database according to BLAST-P searches. Myxo_mac_154 (a 28 residue AMP) had 85% sequence similarity with a transposase (a 592 residue protein) of *M. macrospsorus*, which had associations with several other transposase enzymes, again raising the possibility of acquisition via HGT (Table S3). Three other AMPs, Myxo_mac515, Myxo_mac611, and Myxo_mac628, gave 100% query cover and 100% similarity with three hypothetical proteins of *M. macrosporus*. STRING analysis showed their association with other hypothetical proteins, hydrolases and peptidases (Table S3). In addition, antiSMASH showed the presence of 25 BGCs with possible antibacterial, anticancer, and antioxidant properties.

## *Myxococcus stipitatus* DSM 14,675

Sixty-four putative AMP sequences were extracted from the *M. stipitatus* DSM 14,675 genome, of which eleven were predicted to have good antibacterial activity. In silico analysis predicted three of them to be active against four of the pathogenic bacteria analysed with the other seven having predicted activity against three pathogenic bacteria. Bioinformatic analysis predicted only one AMP was haemolytic against human RBCs, four were predicted to be biofilm active in their native form, and two in mutated forms (Table S2). No putative AMPs were predicted to be active against *S. aureus*, while most were active against *K. pneumoniae*. None of the ‘potent’ AMPs of *M. stipitatus* had high similarity (> 85%) to known proteins by BLAST-P search. Twenty-seven BGCs encoding secondary metabolites were found in the genome of *M. stipitatus* DSM 14,675.

## ***Myxococcus xanthus*** DK 1622 

The *M. xanthus* DK 1622 genome had 82 putative AMP sequences of which 15 were predicted to have activity against three or more pathogenic bacteria. Four out of 15 were predicted to be haemolytic, and one putative AMP (Myxo_xan179) was predicted to be both haemolytic and toxic by the ToxinPred tool. Six were likely to be biofilm active in their native, form while one (Myxo_xan9) was active with a mutation at the 5th amino acid position (Table S2). Myxo_xan210 had 97% sequence similarity (100% query cover) with a hypothetical protein of *M. xanthus* which was functionally associated with several other hypothetical proteins, peptidase S1B family protein, and D-3-phosphoglycerate dehydrogenase (Table S3). There were 23 BGCs identified in the *M. xanthus* DK1622 genome when analysed by the antiSMASH/BAGEL4 tools.

## *Sorangium cellulosum* SoCe56

The *S. cellulosum* SoCe56 genome encoded 114 putative AMP sequences of which 21 were predicted to be active against three or more different bacteria (‘potent’). Although there were not any AMP sequences that were predicted to be active against all five bacteria, 11 of them were predicted to be active against four. Most AMPs were predicted to be active against *K. pneumoniae* (39%), while only 2% were active against *S. aureus*. Of the 21 ‘potent’ AMPs, five were predicted to have haemolytic properties on human erythrocytes by HemoPred, and three of them were predicted to be toxic by ToxinPred, with one predicted to be both haemolytic and toxic. Nine putative AMPs were predicted to be biofilm active in their native form and seven in their mutated forms (Table S2). Upon querying the AMP sequences with BLAST-P, only two exhibited 100% query coverage and > 85% sequence similarity to proteins in the NCBI database. Submitting those proteins to the STRING database revealed functional associations with other proteins. So_ce_56_340, a putative 28 amino acid residue AMP, had 86% similarity (100% query cover) to the ISL3 family transposase of *S. cellulosum* (a 73 amino acid residue protein). This protein was associated with a variety of other proteins that encode putative transposases, recombinases, and hypothetical proteins (Table S3). The peptide being part of the transposase enzyme and associated with other transposases suggests it could have been acquired via mobile genetic elements. The other AMP So_ce_56_913 (a 50-residue peptide) had 98% sequence similarity with a hypothetical (50-residue) protein from *S. cellulosum* which is functionally associated with sce4000, which is a putative hydrolase (Table S3). Profiling *S. cellulosum* SoCe56 genome for biosynthetic gene clusters (BGCs) by ANTISMASH and BAGEL4, 36 BGCs with possible antimicrobial activities were detected.

## *Stigmatella aurantiaca* DW4/3–1

One hundred seven putative AMP sequences were found in the *S. aurantiaca* DW4/3–1 genome with 14 of them predicted to have good activity against three or more pathogenic bacteria. Stig_221 and Stig_926 were predicted to be active against all the five pathogenic bacteria tested, but unfortunately, both were also predicted to be haemolytic to human RBCs, limiting their application value. Forty-six per cent of the putative AMPs were predicted to be active against *K. pneumoniae*. Six of the ‘potent’ putative AMPs in their native form were predicted to have antibiofilm properties along with one in a mutated form (Stig_93) (Table S2).

Stig_715, a 74-residue putative AMP, had 100% similarity with an 88 residue 30S ribosomal protein S20 of *S. aurantiaca*, and Stig_797, a 69-residue, had 99% similarity with a 30S ribosomal protein S18 of *S. aurantiaca*, both of which had functional associations with several other 30S ribosomal proteins and suggesting a house-keeping role. Stig_926 had 99% similarity (100% query cover) with a hypothetical protein of *S. aurantiaca* and was functionally associated with other hypothetical proteins (Table S3). Both Stig_715 and Stig_926 were predicted to have anticancer properties by the ACPred tool. There were 39 BGCs encoding secondary metabolites in the genome.

### Antimicrobial and Antibiofilm Activities

**Fig. 3:**
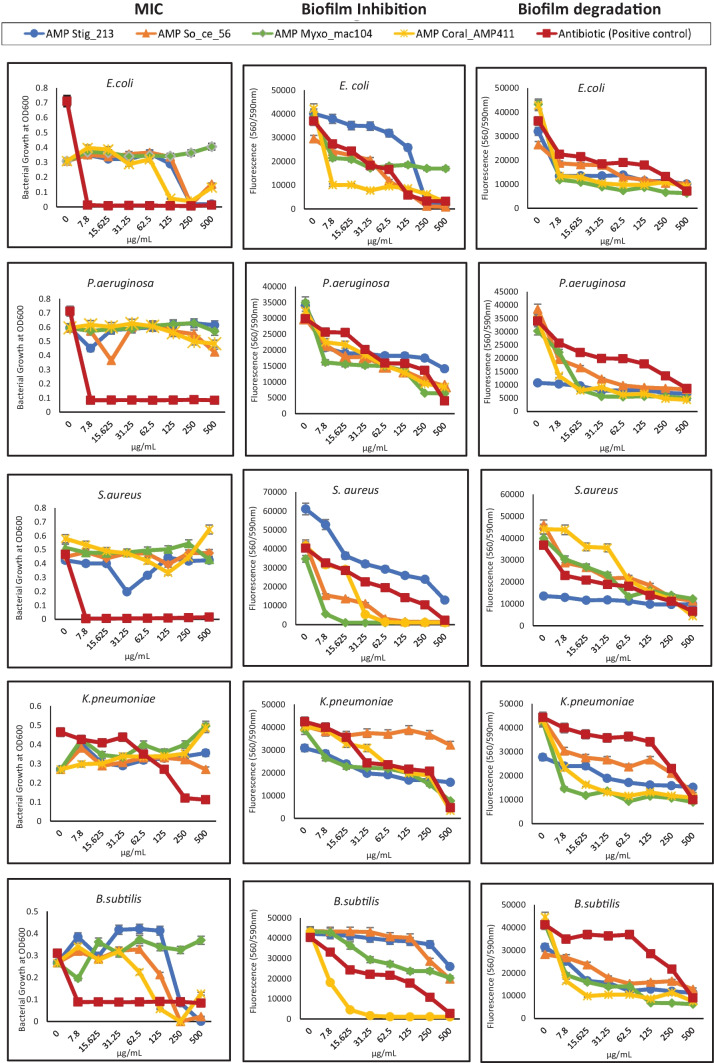
Antimicrobial/antibiofilm activities of synthesized AMPs

Four AMPs were synthesized and tested against five bacterial strains for antimicrobial and antibiofilm activities (Table 3). Comparing the minimum inhibitory concentrations (MIC) of the AMPs to those of the antibiotics revealed that the AMPs were not similarly efficacious. Stig_213 and So_ce_56_913 had a MIC of 250μg/ml, and Coral_AMP411 had a MIC of 125μg/ml against *E. coli*. Against *B. subtilis*, Coral_AMP411 and So_ce_56_913 had a MIC of 250μg/ml. The other organisms were resistant to the AMPs (> 500μg/ml)(Table 3). However, looking at biofilm inhibition assays, Coral_AMP411 and Myxo_mac104 significantly inhibited biofilm formation of *E. coli* and *S. aureus* respectively (*p* < 0.001). With biofilm degradation assays, Coral_AMP411 and Myxo_mac104 significantly reduced bacterial numbers against *E. coli*, *K. pneumoniae,* and *B. subtilis* (*p* < 0.001) (Fig. 3). Coral_AMP411 exhibited a 44.4% reduction in biofilm formation and a 68.8% reduction in biofilm dispersal against *E. coli* at its lowest concentration (7.8 µg/ml). Myxo_mac104, on the other hand, showed a 76.2% reduction in biofilm formation and a 72.6% reduction in biofilm dispersal against *E. coli* at its lowest concentration (7.8 µg/ml) (Table S5).

**Table 3:** MICs for AMPs and antibiotics

	MIC for AMPs and comparator antibiotics $(\mu \mathrm{g}/\mathrm{ml})$							
Organisms	Stig_213	Coral_AMP411	Myxo_mac104	So_ce_56_913	Ampicillin	Piperacillin	Vancomycin	Penicillin
*E. coli*	250	125	> 500	250	7.8	-	-	-
*P. aeruginosa*	> 500	> 500	> 500	> 500	-	7.8	-	-
*S. aureus*	> 500	> 500	> 500	> 500	-	-	7.8	-
*K. pneumoniae*	> 500	> 500	> 500	> 500	250	-	-	-
*B. subtilis*	500	250	> 500	250	-	-	-	7.8

## Discussion

Advances in the understanding of AMP structure/function and the development of AMP-related bioinformatics tools mean that it is now possible to identify putative AMPs in genomes with high confidence [17–19]. In silico approaches can be used to rationally identify a shortlist of candidate AMPs for targeted experimental validation, rather than having to perform expensive and time-consuming screening experiments. Here, we have undertaken the first genome-wide characterization of myxobacterial ribosomally synthesized AMPs and highlight 37 particularly promising candidate AMPs.

AMPs are generally classified according to their source, structure, activities, and amino acid residues [35]. Although mammalian, marine, amphibian, plant, and insect-derived AMPs have been well characterized in the literature, studies on AMPs from microorganisms are limited [35]. Bacteriocins are one group of AMPs obtained from bacteria that have been investigated recently, with probiotic organisms that produce bacteriocins being studied as alternative therapeutic agents [36]. This study analysed eight complete genomes of different myxobacterial species using multiple in silico approaches to screen for potential AMP sequences and predict the properties of putative AMPs. Myxobacteria are well known for their antimicrobial lifestyle and their production of bioactive secondary metabolites, which have been exploited for several decades as therapeutic candidates, with notable success [15, 37]. However, virtually nothing is known about the AMPs produced by myxobacteria, despite the current antibiotic resistance crisis, which demands novel approaches and alternative drug candidates to be investigated. The in silico analyses described here revealed over 600 putative AMP sequences, of which 117 were predicted to have antimicrobial activity against multiple species of bacterial pathogens.

Secondary metabolites of myxobacteria are by and large active primarily against Gram-positive bacteria. For instance, *S. cellulosum* produces several secondary metabolites, including sorangicins, disorazoles, chivosazol, sorangiolids, sulfangolids, etnangien, and thuggacins. These metabolites have been well characterized structurally, and assays of their antimicrobial activities have shown them to have a preponderance of activity against S. *aureus*, yeasts, fungi, and mycobacteria, with limited activity against Gram-negative bacteria [37]. In contrast, the candidate AMP sequences of *S. cellulosum* So ce56 were predicted to have good activity against *K. pneumoniae* (39% of AMPs), *E. coli* (21%), and *P. aeruginosa* (20%), while only 2% were predicted to be active against *S. aureus*. Studies of the predatory activities of live myxobacteria against pathogenic bacteria on non-nutrient agar have demonstrated that the predators kill Gram-negative bacteria like K. *pneumoniae, E. coli*, and *Proteus mirabilis* more effectively than Gram-positive bacteria like *S. aureus* or *Staphylococcus epidermidis* [14]. Therefore, it appears that the mechanisms of secondary metabolite action contrast with those of AMPs, making them worth investigating experimentally. Our in vitro studies highlight that the MICs were not efficacious compared to known antibiotics against the five organisms tested, although some AMPs (Stig_213, Coral_AMP411, and So_ce_56_913) had high MICs $(250\;\mu\mathrm{g}/\mathrm{ml})$ against *E. coli* and *B. subtilis.* A possible reason would be due to the use of a crude product (~ 50% purity) of the synthesized AMP which is a limitation of the study. Therefore, a purified product (> 95%) of the synthesized AMP would give a better perception of their potency. We measured optical densities by spectrophotometry to determine MICs as it is the recommended method in the CLSI/EUCAST guidelines for traditional antibiotics. However, using viable counts to enumerate the colony-forming units after treatment with the AMPs would have allowed differentiation between bacteriostatic and bactericidal properties of the AMPs, which will be important for the characterization of purified AMP products.

AMPs from mammalian sources and insects are naturally anti-inflammatory in addition to their antimicrobial role [38, 39]. All of the 117 ‘potent’ putative AMPs were predicted to be potentially anti-inflammatory, with more than 90% having a probability above 0.6 (Table S2). The APD3 database contains 193 antiviral peptides of which only 12 were from bacterial sources, the rest being from mammalian, amphibian, plant, arthropod, and marine sources. None of the AMP sequences from our study was predicted to have antiviral properties. However, secondary metabolites from myxobacteria have been reported to have potent antiviral activities [40]. Therefore, whether AMPs from myxobacteria generally do not have antiviral properties, or whether further investigations into other myxobacterial genomes are likely to identify AMPs with these properties, is currently unclear. All 117 ‘potent’ putative AMPs were predicted to have antifungal properties, with nine having particularly high prediction scores (> 0.9). This is perhaps to be expected, as fungal cells are abundant in natural environments such as soils, where they are likely to be preyed upon by myxobacteria. Similarly, many secondary metabolites from myxobacteria have antifungal properties, mainly by inhibiting electron flow in the mitochondrial respiratory chain [41].

Cationic amphipathic short peptides (20–50 residues) with high hydrophobicity and net charge are generally good candidates for therapeutic agents, but a lot of them are cytotoxic and fail to be developed further [18]. Cytotoxicity of AMPs is measured by their haemolytic properties on human erythrocytes and killing of lymphocytes [42]. Only four putative AMP sequences from *S. cellulosum* and one from *M. xanthus* were predicted to be cytotoxic, while 26 of the 672 putative AMP sequences across the eight genomes were predicted to be haemolytic. Several AMPs, predominantly from mammalian sources, have been reported to have anticancer properties by acting on the negatively charged phosphatidylserine moieties of cancer cell membranes [43]. Anaero2CP1_256, Anaero2CP1_262, Anaero2CP1_264 (from *A. dehalogenans*), Haliangium_och1055 (from *H. ochraceum*), Stig_715, and Stig_926 (from *S. aurantiaca*) are proposed to have anticancer properties. Stig_715 in particular, a 75-residue peptide with a molecular weight of 6822, had a high prediction score (0.972) and should be prioritized for investigating further.

The pathogenesis of many infectious diseases is related to biofilm formation, which allows pathogens to escape the effect of antibiotics and immune system mechanisms [44]. Therefore, there is a need for drugs that can disrupt biofilms and kill the sessile bacteria therein, rather than just killing planktonic cells. Several AMPs reported in the literature can prevent biofilm formation, downregulating the genes encoding the quorum sensing factors which stimulate biofilm formation, or degrading preformed biofilms [44]. Fifty five of the 117 ‘potent’ myxobacterial putative AMPs were predicted to have antibiofilm properties with eight of them having particular high prediction scores (> 0.9). Of the remaining 62, 47 were not predicted to have antibiofilm properties in their native form, but 15 of them could be mutated at various amino acid residue positions resulting in positive predictions. Therefore, there are some interesting candidate AMPs potentially produced by myxobacteria that seem likely to be able to kill not only planktonic bacteria but also those embedded in biofilms, and they should be prioritized for experimental validation. Our in vitro antibiofilm assays showed some promising results despite the MICs not being very efficacious. Biofilm inhibition and degradation assays showed that Coral_AMP411 and Myxo_mac104 had good antibiofilm activity against *E.coli*, *K.pneumoniae*, and *B.subtilis* compared to known antibiotics. Again, using a purified product of the synthesized AMP will reveal insights about the antibiofilm activities of the AMPs. Although synthesizing these AMPs is expensive compared to traditional antibiotics, if their efficacy is better, the cost can be supported in the global crisis of antimicrobial resistance. Myxobacteria utilize several mechanisms as part of their predatory activity, including secretion of secondary metabolites, digestion with hydrolytic enzymes, and contact-dependent killing [45]. Studies have also shown that secreted proteins and contact-dependent killing are selective towards particular target bacteria [46]. In our study, AMPs were predicted to have particularly good activity against Gram-negative organism, an activity reported by Arend et al. to be associated with cell contact dependent killing [46]. This suggests that it would be beneficial to investigate the functional roles of AMPs. To elucidate whether AMPs interact with other predatory processes, the myxobacterial potent AMPs were searched against the NCBI database using BLAST-P, and STRING employed to suggest functional annotations and associations. Of the 14 AMPs that had > 85% similarity (100% query cover) with known proteins, nine of them were found within hypothetical proteins, which were functionally associated with several other hypothetical proteins, which is encouraging as the novelty of such proteins could potentially be exploited for innovative therapeutics.

Two ‘potent’ putative AMPs, Myxo_ful154 and So_ce_56_340, had sequence similarity with transposase enzymes, suggesting they could have been acquired via mobile genetic elements and lateral transfer. Conversely, Stig_715 exhibited sequence similarity to the 30S ribosomal protein S20, which would be expected to have been transmitted vertically. Presumably, contemporary sets of AMPs will have been acquired from both mobile genetic elements and by linear descent. Supporting this interpretation, pangenome analysis of *C. coralloides* genomes showed that 25% (12/48) of AMP sequences were part of the core genome, but the majority were found in the accessory genome. A recent pangenome study of *Corallococcus* spp. isolates gave similar results [47], finding that 30% of genes within each genome formed the core genome, with 70% of each genome belonging to the accessory genome. In contrast, the same study found that only 10% of BGCs belonged to the core *Corallococcus* genome [47], suggesting that AMPs may be more highly conserved than BGCs in myxobacterial genomes, an observation which merits further study.

To further shortlist those putative AMPs with the greatest likelihood of potential application in the clinic, the 117 ‘potent’ putative AMPs were checked against CAMPR3 predictions and filtered according to predicted toxicity, length, charge, and hydrophobicity (Table 2). We propose that these sequences are particularly worthy of being synthesized and tested for activity against pathogenic organisms using purified products of the AMPs.

## Conclusions

The in silico survey of eight complete myxobacterial genomes presented here revealed 672 AMPs with possible antimicrobial activities, of which 117 were potent AMPs. Further analysis of the 117 potent AMPs using bioinformatic tools has filtered out the 37 best AMPs that warrant synthesis and experimental testing in the laboratory. A selected subset of predicted AMPs was synthesized and shown to have antimicrobial and antibiofilm activities in vitro, supporting the validity of in silico screening approaches prior to experimental validation. Further validation and characterization of candidate myxobacterial AMPs in the laboratory would be worthwhile, as many were also predicted to possess anti-inflammatory and antifungal properties which would be beneficial if developed as therapeutics.

## Supplementary Information

Below is the link to the electronic supplementary material.

Supplementary file1 (XLSX 55 KB)

Supplementary file2 (XLSX 192 KB)

Supplementary file3 (DOCX 22 KB)

Supplementary file4 (XLSX 38 KB)

Supplementary file5 (XLSX 25 KB)

## Data Availability

Most data generated or analysed during this study are included in this published article and its supplementary information files. Those datasets generated during and/or analysed during the current study that are not published here are available from the corresponding author on reasonable request.
