# Supplementary material for: In Silico and In Vitro Analyses Reveal Promising Antimicrobial Peptides from Myxobacteria
Source: Probiotics Antimicrob Proteins. 2022 Dec 31;15(1):202–14. doi: 10.1007/s12602-022-10036-4 (PMC9839799; doi:10.1007/s12602-022-10036-4)
Supplement: Supplementary file 3 — Supplementary file3 (DOCX 22 KB) [file 12602_2022_10036_MOESM3_ESM.docx]

| **AMP** | **Proteins that had highest similarity** | **Associated Protein Identifiers** | **Functional Annotations** |
| --- | --- | --- | --- |
| Anaero2CP1_10 | WP_106392650.1 tetratricopeptide repeat protein [*Enhygromyxa salina*] | Adeh_1709 | Hypothetical protein |
|  |  | Adeh_1711 | Hypothetical protein |
|  |  | Adeh_3691 | Hypothetical protein |
|  |  | Adeh_1710 | Hypothetical protein |
|  |  | Adeh_1712 | Hypothetical protein |
|  |  | Adeh_2033 | Hypothetical protein |
|  |  | Adeh_2929 | Hypothetical protein |
|  |  | Adeh_3450 | Hypothetical protein |
|  |  | Adeh_3650 | Hypothetical protein |
|  |  | Adeh_4322 | Hypothetical protein |
| Anaero2CP1_341 | ACL64149.1 hypothetical protein [*Anaeromyxobacter dehalogenans* 2CP-1] | Adeh_0028 | Transcriptional regulator, lysr family; |
|  |  | Adeh_0745 | Hypothetical protein |
|  |  | Adeh_0542 | Lysr family transcriptional regulator |
|  |  | Adeh_1034 | Lysr family transcriptional regulator |
|  |  | Adeh_0817 | Hypothetical protein |
|  |  | Adeh_2267 | Hypothetical protein |

**Table 1: Functional STRING analysis on *Anaeromyxobacter dehalogenans* AMP sequences Anaero2CP1_10 and Anaero2CP1_341.**

| **AMP** | **Proteins that had highest similarity** | **Associated Protein Identifiers** | **Functional Annotations** |
| --- | --- | --- | --- |
| Haliangium_och974 | WP_012825848.1 hypothetical protein [*Haliangium ochraceum*] | Hoch_0583 | Hypothetical protein |
|  |  | Hoch_0584 | Hypothetical protein |
|  |  | Hoch_0585 | Hypothetical protein |
|  |  | Hoch_0582 | Hypothetical protein |
| Haliangium_och996 | WP_012826197.1 hypothetical protein [*Haliangium ochraceum*] | Hoch_0976 | Hypothetical protein |
|  |  | Hoch_0975 | Hypothetical protein |
|  |  | Hoch_0974 | Hypothetical protein |

**Table 2: Functional STRING analysis on *Haliangium ochraceum* AMP sequences Haliangium_och974 and Haliangium_och996.**

| **AMP** | **Proteins that had highest similarity** | **Associated Proteins Identifier** | **Functional Annotations** |
| --- | --- | --- | --- |
| Myxo_mac154 | ATB51294.1 transposase [*Myxococcus macrosporus* DSM 14697] | AEI69173.1 | Transposase, is66 family protein |
|  |  | AEI65356.1 | Transposase, is66 orf2 like protein |
|  |  | AEI68324.1 | Putative transposition helper protein, IS66 |
|  |  | AEI68319.1 | Putative transposase |
|  |  | AEI68734.1 | Transposase is66 |
|  |  | ruvA | The RuvA-RuvB helicase complex |
|  |  | uvrB | Exonuclease abc subunit b |
|  |  | AEI69172.1 | Transposase, is66 family protein; |
|  |  | ruvB | The RuvA-RuvB helicase complex |
|  |  | ruvC | Crossover junction endodeoxyribonuclease |
|  |  | AEI65355.1 | Transposase, is66 family protein |
| Myxo_mac515 | AEI63105.1 hypothetical protein [*Myxococcus macrosporus*] | AEI63105.1 | Hypothetical protein |
|  |  | AEI63104.1 | Hypothetical protein |
| Myxo_mac611 | WP_013938303.1 hypothetical protein [*Myxococcus macrosporus*] | A176_05562 | Hypothetical protein |
|  |  | A176_05563 | Proline aminopeptidase protein |
| Myxo_mac628 | AEI62446.1 hypothetical protein[*Myxococcus macrosporus*] | AEI62446.1 | Hypothetical protein |
|  |  | AEI62447.1 | Transcriptional regulators |
|  |  | AEI62445.1 | Serine/threonine protein kinase |
|  |  | AEI62444.1 | Hypothetical protein |

**Table 3: Functional STRING analysis on *Myxococcus macrosporus* AMP sequences Myxo_mac154, Myxo_mac515, Myxo_mac611, Myxo_mac628**

| **AMP** | **Proteins that had highest similarity** | **Associated Proteins Identifiers** | **Functional Annotations** |
| --- | --- | --- | --- |
| So_ce_56_340 | WP_049876229.1 ISL3 family transposase [*Sorangium cellulosum*] | sce2108 | Putative transposase |
|  |  | sce2109 | Hypothetical protein |
|  |  | sce6650 | Recombinase |
|  |  | sce0272 | Hypothetical protein |
|  |  | sce2110 | Hypothetical protein |
|  |  | sce0273 | Hypothetical protein |
| So_ce_56_913 | CAN94159.1 hypothetical protein [*Sorangium cellulosum*] | sce3999 | Hypothetical protein |
|  |  | sce4000 | Putative hydrolase |
|  |  | sce3998 | Unnamed protein product |

**Table 4: Functional STRING analysis on *Sorangium cellulosum* AMP sequences So_ce_56_340 and So_ce_56_913**

| **AMP** | **Proteins that had highest similarity** | **Associated Proteins Identifiers** | **Functional Annotations** |
| --- | --- | --- | --- |
| Myxo_xan210 | ABF90104.1 hypothetical protein [*Myxococcus xanthus* DK 1622] | MXAN_6353 | Hypothetical protein |
|  |  | MXAN_6352 | Peptidase S1B family |
|  |  | MXAN_6354 | Hypothetical protein |
|  |  | MXAN_6355 | Hypothetical protein |
|  |  | serA | D-3-phosphoglycerate dehydrogenase |
|  |  | MXAN_6351 | Hypothetical protein |

**Table 5: Functional STRING analysis on *Myxococcus xanthus* AMP sequences Myxo_xan210**

| **AMP** | **Proteins that had highest similarity** | **Associated Protein Identifiers** | **Functional Annotations** |
| --- | --- | --- | --- |
| Stig_797 | ADO69193 30S ribosomal protein S18 [*Stigmatella aurantiaca*] | rpsR | Small subunit ribosomal protein s18 |
|  |  | rpsI | Small subunit ribosomal protein s9 |
|  |  | rpsL | Small subunit ribosomal protein s12 |
|  |  | rpsG | Small subunit ribosomal protein s7 |
|  |  | rpsJ | Small subunit ribosomal protein s10 |
|  |  | rpsS | Small subunit ribosomal protein s19 |
|  |  | rpsC | Small subunit ribosomal protein s3 |
|  |  | rpsQ | Small subunit ribosomal protein s17 |
|  |  | rpsH | Small subunit ribosomal protein s8 |
|  |  | rpsE | Small subunit ribosomal protein s5 |
|  |  | rpsM | Small subunit ribosomal protein s13 |
| Stig_715 | WP_002616933.1 30S ribosomal protein S20 [*Stigmatella aurantiaca*] | rpsT | Hypothetical protein |
|  |  | rpmA | Large subunit ribosomal protein l27 |
|  |  | rpsI | Small subunit ribosomal protein s9 |
|  |  | rpsL | Small subunit ribosomal protein s12 |
|  |  | rpsG | Small subunit ribosomal protein s7 |
|  |  | rpsJ | Small subunit ribosomal protein s10 |
|  |  | rpsS | Small subunit ribosomal protein s19 |
|  |  | rpsC | Small subunit ribosomal protein s3 |
|  |  | rpsQ | Small subunit ribosomal protein s17 |
|  |  | rpsH | Small subunit ribosomal protein s8 |
|  |  | rpsE | Small subunit ribosomal protein s5 |
| Stig_926 | WP_002613330.1 hypothetical protein [*Stigmatella aurantiaca*] | STAUR_0358 | Hypothetical protein |
|  |  | STAUR_7872 | Hypothetical protein |
|  |  | STAUR_0359 | Hypothetical protein |
|  |  | STAUR_2385 | Hypothetical protein |
|  |  | STAUR_0360 | Hypothetical protein |

**Table 6: Functional STRING analysis on *Stigmatella aurantiaca* AMP sequences Stig_797, Stig_715 and Stig_926**
